# Datasets on the statistical properties of the first 3000 squared positive integers

**DOI:** 10.1016/j.dib.2017.09.055

**Published:** 2017-10-06

**Authors:** Hilary I. Okagbue, Muminu O. Adamu, Pelumi E. Oguntunde, Abiodun A. Opanuga, Ayodele A. Adebiyi, Sheila A. Bishop

**Affiliations:** a Department of Mathematics, Covenant University, Canaanland, Ota, Nigeria; b Department of Mathematics, University of Lagos, Akoka, Lagos, Nigeria; c Department of Computer and Information Sciences, Covenant University, Canaanland, Ota, Nigeria

**Keywords:** Positive integer, Digits sum, Harrell-Davis quantiles, Boxplots, Bootstrap, M-estimators, Confidence intervals, Curve estimation, Model fit

## Abstract

The data in this article are as a result of a quest to uncover alternative research routes of deepening researchers’ understanding of integers apart from the traditional number theory approach. Hence, the article contains the statistical properties of the digits sum of the first 3000 squared positive integers. The data describes the various statistical tools applied to reveal different statistical and random nature of the digits sum of the first 3000 squared positive integers. Digits sum here implies the sum of all the digits that make up the individual integer.

**Specifications Table**

**Table:** 

Subject area	Mathematics
More specific subject area	Number Statistics, Computational number theory
Type of data	Tables and Figures
How data was acquired	The raw data is available in mathematical literature
Data format	Analyzed
Experimental factors	Zero and negative integers were not considered
Experimental features	Exploratory data analysis, mathematical computation
Data source location	Covenant University Mathematics Laboratory, Ota, Nigeria
Data accessibility	All the data are in this data article

**Value of the data**

• The data provides the exploratory statistics of digits sum of squared positive integers and their subsets.
• This technique of analysis can be used in data reduction.
• The data analysis can be applied to other known numbers.
• The data when completely analyzed can help deepen the understanding of the random nature of integers.

## 1 Data

**Fig. 1:**
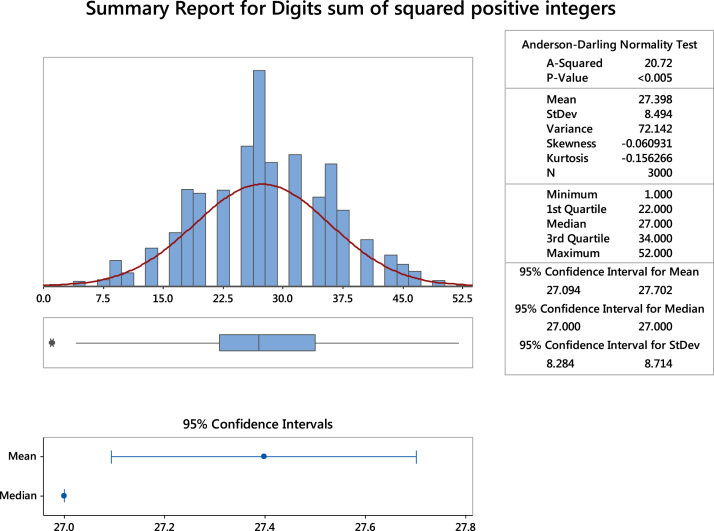
The summary statistics of the digits sum of squared positive integers. **Remark**: The gaps observed in the histogram are because the digits sum of squared positive integers cannot yield some numbers such as: 2, 3, 5, 6, 8, 11, 12, 14, 15 and so on.

**Fig. 2:**
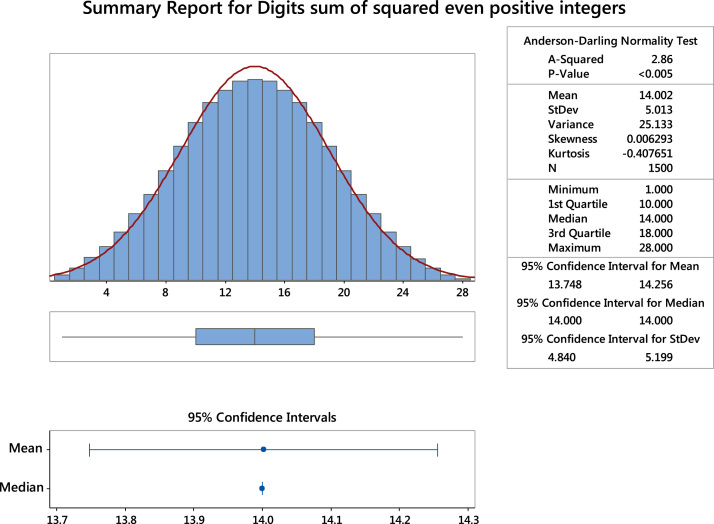
The summary statistics of the digits sum of squared even positive integers. **Remark**: It can be seen that the mean and median of the data set are almost the same.

**Fig. 3:**
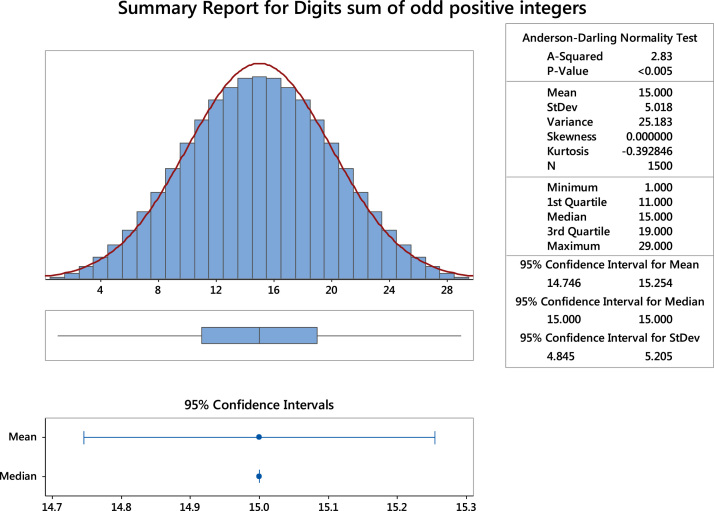
The summary statistics of the digits sum of squared odd positive integers. **Remark**: Here, the mean and median of the data set are the same.

The data provides a description of the statistical properties of the digits sum of the first 3000 squared positive integers and the subsets. The subsets are the even and odd positive integers. The subsets are equivalence and their descriptive statistics are summarized in Fig. 1, Fig. 2, Fig. 3**:**

## 2 Experimental design, materials and methods

The digits sum or digital sum of integers has been a subject of interest because of its application in cryptography, primality testing, random number generation and data reduction. Details on the origin, theories and applications of the digits sum of squared positive integers, integers and other important number sequences can be found in [1], [2], [3], [4], [5], [6], [7], [8], [9], [10], [11], [12], [13], [14], [15], [16], [17], [18], [19], [20], [21], [22], [23], [24], [25], [26], [27], [28]. Recently digits sum and digital root have been applied in the analysis of lotto results [29].

### 2.1 Exploratory data analysis

**Fig. 4:**
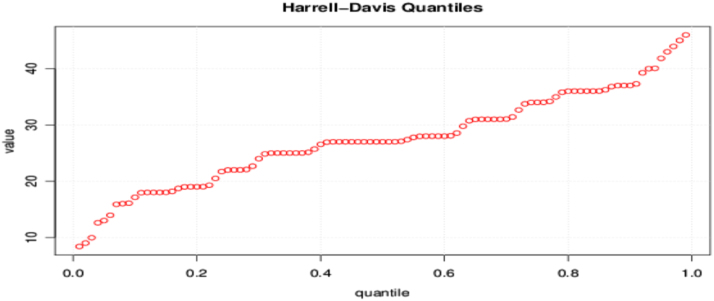
Harrell-Davis quantiles.

The true nature of the percentiles are shown using the Harrell-Davis quantile which is a better estimator and a measure of variability because it makes use of the data in totality rather than the percentiles that are based on order statistics. The Harrell-Davis quantile of the digits sum of square of positive integers is shown in Fig. 4.

Bootstrap methods are useful in construction of highly accurate and reliable confidence intervals (C.Is) for unknown and complicated probability distributions. The data for was resampled many times and C.Is was generated for the mean and the standard deviation. Bootstrap results varied slightly with the observed mean and standard deviation and convergence occurs as the confidence level increases. These are shown in Table 1, Table 2:

**Table 1:** The bootstrap confidence interval for the mean of the digits sum of square of positive integers.

Confidence level (%)	Lower limit	Upper limit
99	27.02	27.76
98	27.03	27.77
97	27.07	27.75
96	27.08	27.72
95	27.10	27.70
94	27.12	27.68
93	27.12	27.70
92	27.12	27.66
91	27.12	27.66
90	27.14	27.64

**Table 2:** The bootstrap confidence interval for the standard deviation of the digits sum of square of positive integers.

Confidence level (%)	Lower limit	Upper limit
99	8.22	8.763
98	8.246	8.735
97	8.262	8.715
96	8.261	8.709
95	8.292	8.700
94	8.308	8.693
93	8.29	8.689
92	8.325	8.681
91	8.316	8.66
90	8.311	8.674

The bootstrap estimate of the mean is closed to the observed one. However, the median remained unchanged. This is an evidence of the robustness and the resistant nature of the median against undue influence of outliers. This is also in agreement with the bootstrap confidence limits. The summary is shown in Table 3.

**Table 3:** Estimation results of bootstrap of the mean and median of digits sum of squared positive integers.

Statistic	P1	P5	Q1	Q2 (estimate)	Q3	P95	P99	S.D.	I.Q.R.
Mean	27.039	27.14	27.278	27.398	27.487	27.639	27.712	0.15221	0.20933
Median	27	27	27	27	27	27	27	0	0

P1=first\ percentile,P5=fifth\ percentile,Q1=first\ quartile,Q2=second\ quartile\ or\ the\ estimate,Q3=third\ quartile,P95=ninety\ five\ percentile,P99=ninety\ nine\ percentile,S.D.=standard\ deviation,I.Q.R.=the\ inter\ quartile\ range.

The M-Estimators are checked for the convergence to the mean or the median. The M-Estimators are robust and resistant to the undue effect of outliers. Technically, an M-Estimator can be assumed as the fixed point of the estimating function. The results of the M-estimator for the digits sum of the first 3000 squared positive integer is summarized in Table 4.

**Table 4:** The M-estimators for the first 3000 squared positive integers.

	Huber's M-estimator^a^	Tukey's biweight^b^	Hampel's M-estimator^c^	Andrews' wave^d^
Sum of the digits of the squared positive integer.	27.43	27.44	27.42	27.44

**Remark**: The three M-estimators are the same but are closer to the mean than the median. This is an indication of the irregular behavior of the distribution.

a The weighting constant is 1.339.

b The weighting constant is 4.685.

c The weighting constants are 1.700, 3.400, and 8.500.

d The weighting constant is 1.340*pi.

**Fig. 5:**
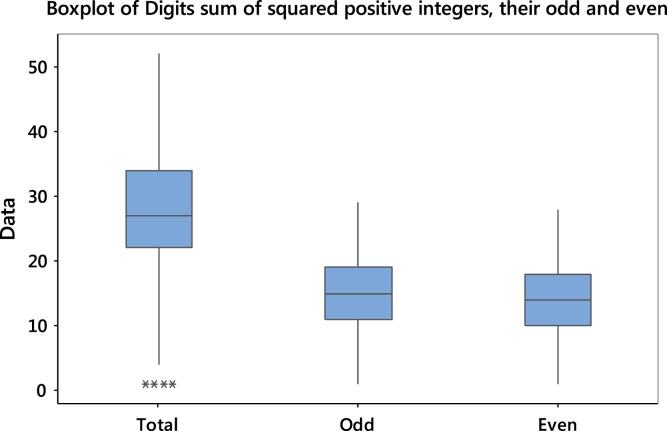
Boxplot summary of the digits sum of the first 3000 squared positive integers.

The boxplot is an exploratory data analysis tool used to display graphically, the quantiles of a given numerical data. Outliers or extreme values are easily precipitated from the data and displayed graphically. The boxplots of the digits sums of squared positive integers and their subsets are shown in Fig. 5:

The data is slightly skewed to the left for the three cases with some outliers appearing in the case of the total. As the sample size increases, the frequency of the occurrence of the numbers below mean reduces and more outliers can also be obtained. On the other hand, more numbers are expected to appear as the sample size increases.

**Fig. 6:**
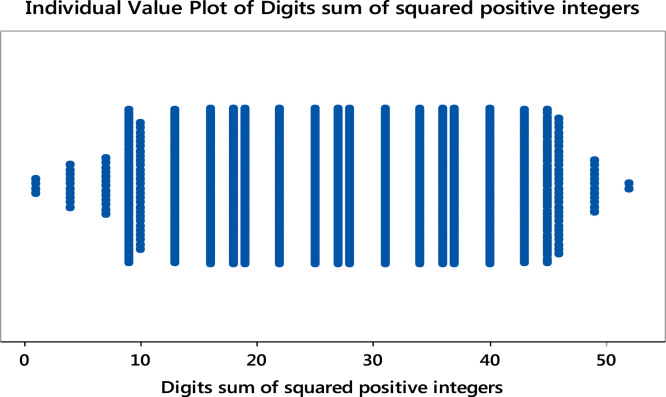
Individual value plot of digits sum of the first 3000 squared positive integers. **Remark**: Some gaps in the plot are synonymous with the result of the histogram. Some extreme values are also noticed in the plot.

**Fig. 7:**
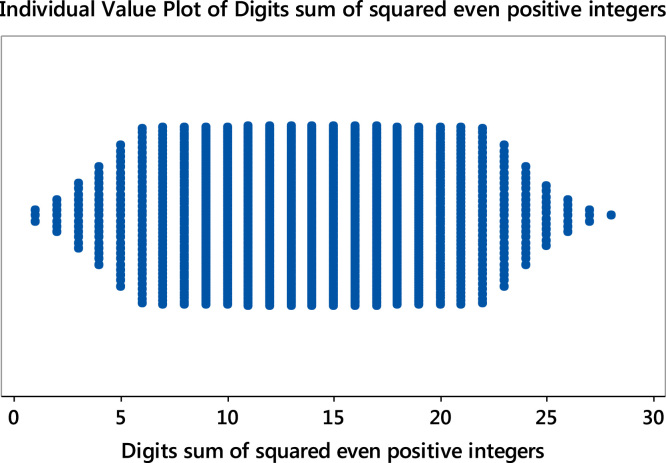
Individual value plot of digits sum of the squared even positive integers.

**Fig. 8:**
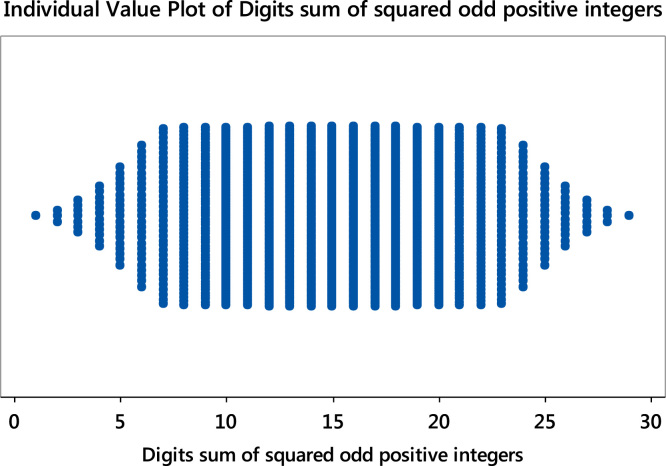
Individual value plot of digits sum of the squared odd positive integers. **Remark**: The plots for the even and odd are identical.

Particular patterns can be depicted through the use of individual value plots of observations. Some unique patterns were obtained for the even, odd and total squared positive integers. This is shown in Fig. 6, Fig. 7, Fig. 8**:**

**Fig. 9:**
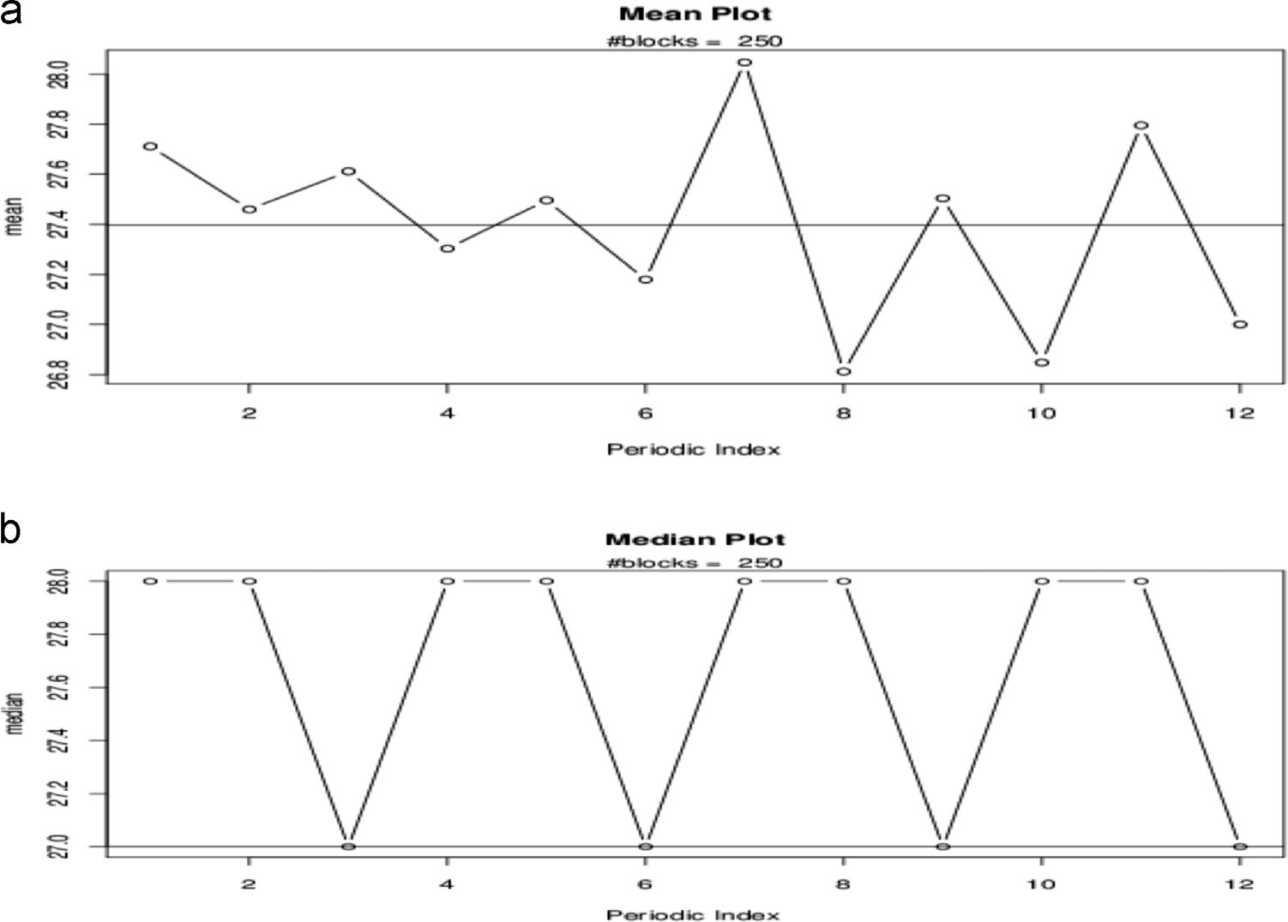
**a:** The mean plot, **b:** The median plot.

The mean plot and median plot are shown in Fig. 9**a** and **b.**

The mean plot showed the behavior of the mean. This is almost the same result by the bootstrap and bootstrap confidence intervals. As excepted the median plot is an indication of the robustness of the median.

**Fig. 10:**
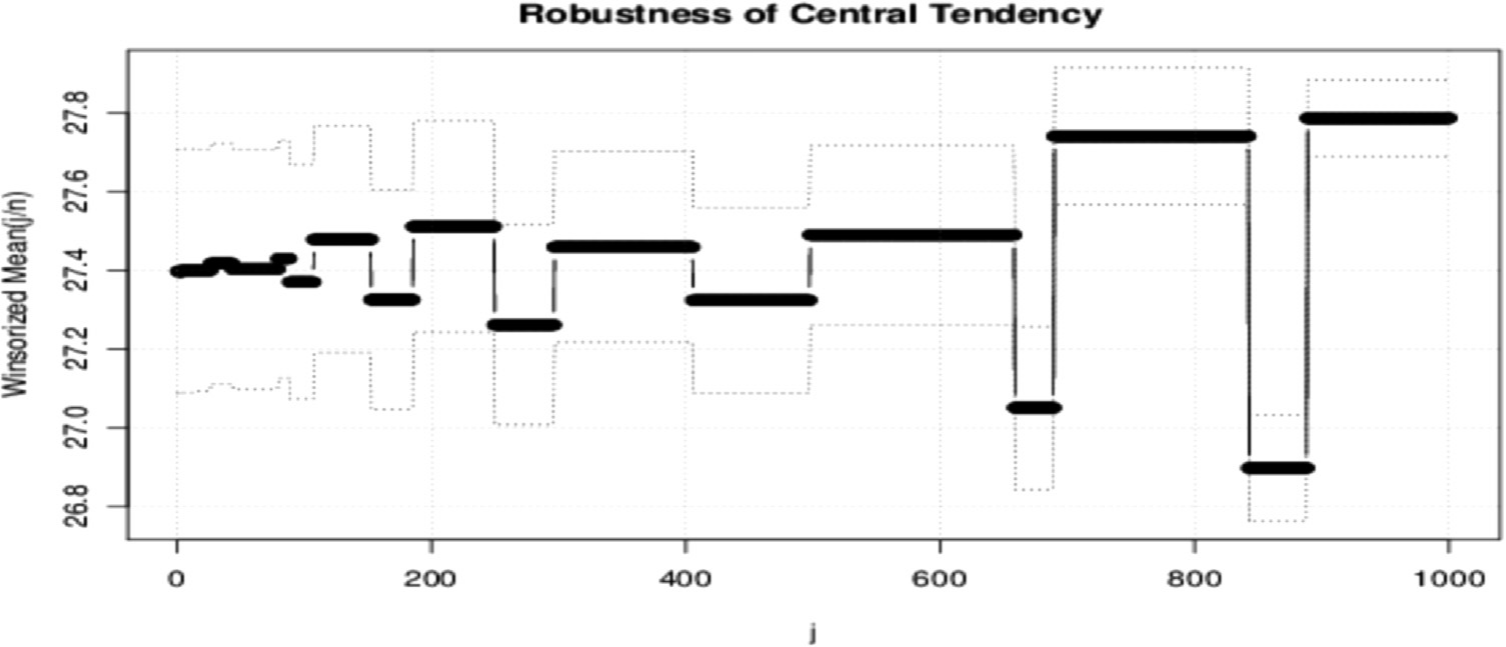
The Winsorized mean and robustness.

**Fig. 11:**
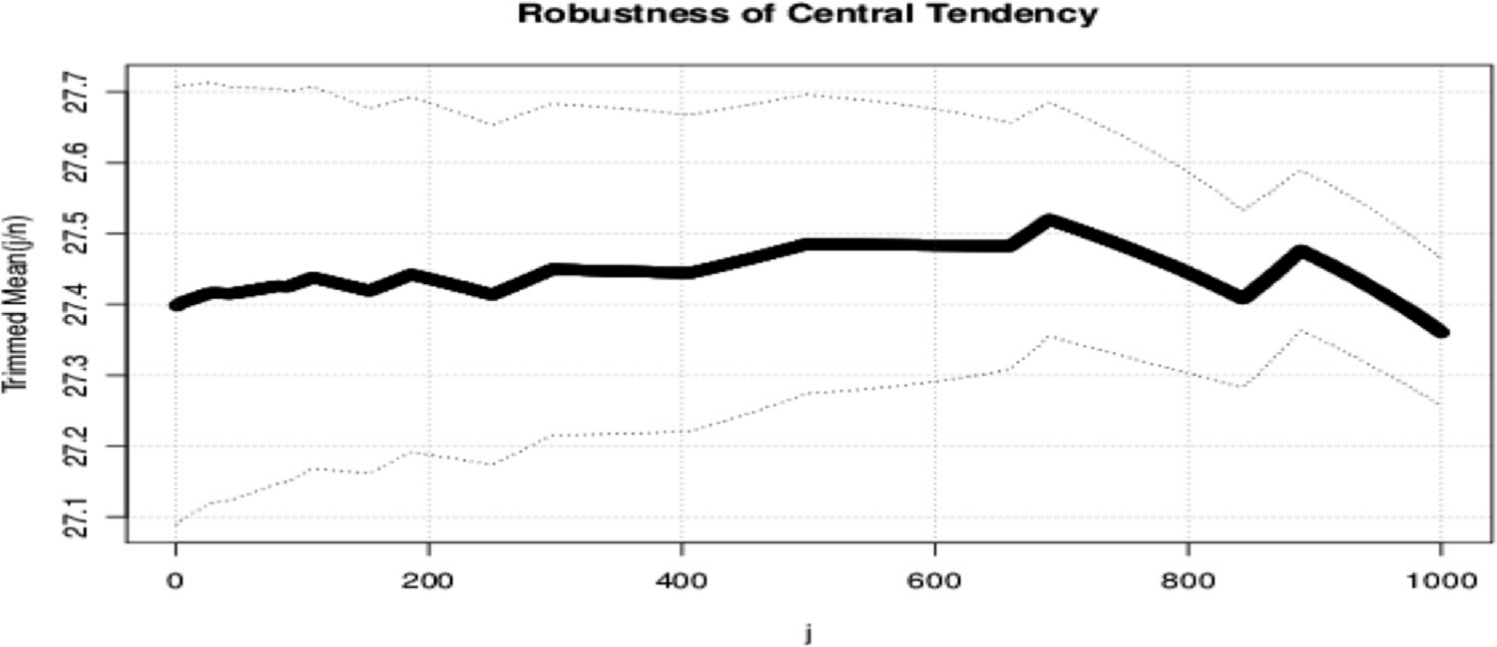
The trimmed mean and robustness.

Winsorizing and trimming are two ways of achieving robustness. The robustness of the central tendency (mean) of the digits sum of the first 3,000 squared positive integers was considered. These are shown in Fig. 10, Fig. 11.

The data is robust because the possibility of obtaining outliers or extreme values decreases as more values are expected to cluster around the mean. As the sample size increases, the extreme values become fewer. In the case of trimming, the same result is obtained since there are few extreme values to exclude from the analysis.

### 2.2 Curve estimation

There are few curve estimation models that are available in fitting a given data. The result of fitting the digits sum of the first 3000 squared positive integers using the models is shown in Table 5.

**Table 5:** Model fit for the digits sum of the first 3000 squared positive integers.

Model	R	Adjusted/R square	SE of the estimates
Linear	0.466	0.217	7.516
Logarithmic	0.488	0.238	7.416
Inverse	0.179	0.032	8.359
Quadratic	0.472	0.223	7.489
Cubic	0.493	0.243	7.392
Compound	0.442	0.195	0.341
Power	0.516	0.266	0.326
S	0.300	0.090	0.363
Growth	0.442	0.195	0.341
Exponential	0.442	0.195	0.341

**Remark**: The low values of the R and adjusted R square indicate that the models barely fit the data and can give misleading results when used in prediction. Moreover, the power model provides the best fit and the inverse model provides the worst fit.

### 2.3 Probability distribution fit

Digits sum of the first 3000 squared positive integers is best fitted by Cauchy distribution and the details are shown in Table 6. This was done using EasyFit software.

**Table 6:** Summary of the data fit.

Parameter	Estimated value	Standard deviation
Location	27.31296	0.129009
Scale	4.85180	0.122221

**Remark**: The data exhibits the characteristics of Cauchy distribution; the goodness of fit (Kolmogorov-Smirnov) test showed the statistic of 0.08616.

### 2.4 Mathematical computational results

The raw data of sum of the digits square of the first 3000 integers can be used to generate another set of numbers by finding the absolute value of the difference of two consecutive numbers and the total data generated is the initial data minus 1. The process was repeated until the mode and the median was equal to one. This is because any further step(s) add little or no effect to the analysis and also to save computational time. Normality is reduced by the process as evidenced by the increase in kurtosis and skewness. This is shown in Table 7.

**Table 7:** Summary of the mathematical computation result.

Data	Count	Sum	Average	Variance	Median	Mode	Kurtosis	Skewness
Raw	3000	82193	27.39767	72.15358	27	27	−0.156	−0.06
1	2999	19446	6.484161	22.55003	6	2	1.228	0.988
2	2998	16061	5.357238	17.64978	4	2	2.196	1.278
3	2997	11968	3.993327	10.51798	4	5	4.045	1.563
4	2996	9012	3.008011	10.12681	2	1	4.740	1.905
5	2995	7218	2.410017	6.214596	2	1	7.225	2.235
6	2994	6173	2.06179	6.199655	1	1	7.410	2.312
7	2993	5261	1.757768	4.47105	1	1	10.190	2.618
8	2992	5140	1.717914	4.635881	1	1	10.377	2.711
9	2991	3949	1.320294	2.744869	1	1	13.765	2.954
10	2990	3831	1.281271	2.979743	1	1	14.716	3.098
